# Novel human mutation and CRISPR/Cas genome-edited mice reveal the importance of C-terminal domain of MSX1 in tooth and palate development

**DOI:** 10.1038/srep38398

**Published:** 2016-12-05

**Authors:** Silvia Naomi Mitsui, Akihiro Yasue, Kiyoshi Masuda, Takuya Naruto, Yoshiyuki Minegishi, Seiichi Oyadomari, Sumihare Noji, Issei Imoto, Eiji Tanaka

**Affiliations:** 1 Department of Orthodontics and Dentofacial Orthopedics, Institute of Biomedical Sciences, Tokushima University Graduate School, 3-18-15 Kuramoto-cho, Tokushima 770-8504, Japan; 2 Division of Molecular Biology, Institute of Advanced Enzyme Research, Tokushima University, 3-18-15 Kuramoto-cho, Tokushima 770-8503, Japan; 3 Department of Human Genetics, Institute of Biomedical Sciences, Tokushima University Graduate School, 3-18-15 Kuramoto-cho, Tokushima 770-8503, Japan; 4 Division of Molecular Medicine, Institute of Advanced Enzyme Research, Tokushima University, 3-18-15 Kuramoto-cho, Tokushima 770-8503, Japan; 5 Tokushima University, 2-24 Shinkura-cho, Tokushima 770-8501, Japan

## Abstract

Several mutations, located mainly in the MSX1 homeodomain, have been identified in non-syndromic tooth agenesis predominantly affecting premolars and third molars. We identified a novel frameshift mutation of the highly conserved C-terminal domain of MSX1, known as Msx homology domain 6 (MH6), in a Japanese family with non-syndromic tooth agenesis. To investigate the importance of MH6 in tooth development, *Msx1* was targeted in mice with CRISPR/Cas system. Although heterozygous MH6 disruption did not alter craniofacial development, homozygous mice exhibited agenesis of lower incisors with or without cleft palate at E16.5. In addition, agenesis of the upper third molars and the lower second and third molars were observed in 4-week-old mutant mice. Although the upper second molars were present, they were abnormally small. These results suggest that the C-terminal domain of MSX1 is important for tooth and palate development, and demonstrate that that CRISPR/Cas system can be used as a tool to assess causality of human disorders *in vivo* and to study the importance of conserved domains in genes.

Non-syndromic tooth agenesis has been associated with mutations in a variety of genes involved in tooth morphogenesis. Among them, mutations in *MSX1* and *PAX9* have been reported in premolar and molar agenesis, respectively^1^,^2^,^3^. Additionally, *AXIN2, WNT10A*, and *EDA* mutations produce severe tooth agenesis of variable patterns^4^,^5^,^6^.

The *MSX* homeobox gene family encodes transcription factors that play important roles in inductive tissue interaction during vertebrate development^7^,^8^. *Msx1* is expressed in developing limb buds and craniofacial structures, including the neural crest, branchial arches, and sensory placodes^9^,^10^. It is markedly expressed in the dental papilla and follicle during the cap stage, declining progressively in the bell stage^11^. The MSX1 protein has seven ancient and highly conserved Msx homology (MH) domains that act as binding or functional domains^12^. In humans, *MSX1* mutations have also been associated with syndromic tooth agenesis in Wolf-Hirschhorn syndrome, Witkop syndrome, and orofacial clefting^13^,^14^,^15^. Furthermore, *MSX1* mutations are present in 2% of non-syndromic cleft lip and palate cases^16^,^17^.

The availability of limited number of cases or families, reduced penetrance and locus heterogeneity are some of the factors limiting traditional strategies to identify disease mutations^18^. Although, with the advert of next-generation sequencing platforms, part of the limitations have been possible to overcome with proper strategies, biological validation is indispensable to assess the causality of uncharacterized variants and genes, especially when is identified in a single family^19^.

Animal models have contributed greatly to elucidate the molecular and developmental mechanisms of human diseases as well as potential therapeutic targets. Meanwhile, transcription activator-like effector nucleases (TALENs) and the clustered regularly interspaced short palindromic repeat (CRISPR)/CRISPR-associated protein (Cas) system enable efficient and precise genome editing^20^,^21^. These techniques have been used to produce gene mutations in diverse organisms and thereby to assess the causality of human mutations in animal models^22^,^23^,^24^.

Here, we report a novel tooth agenesis-causing mutation in the C-terminus of MSX1. Genotype-phenotype relation was analysed through CRISPR/Cas-mediated genome editing in mice.

## Results

### Clinical data

**Figure 1:**
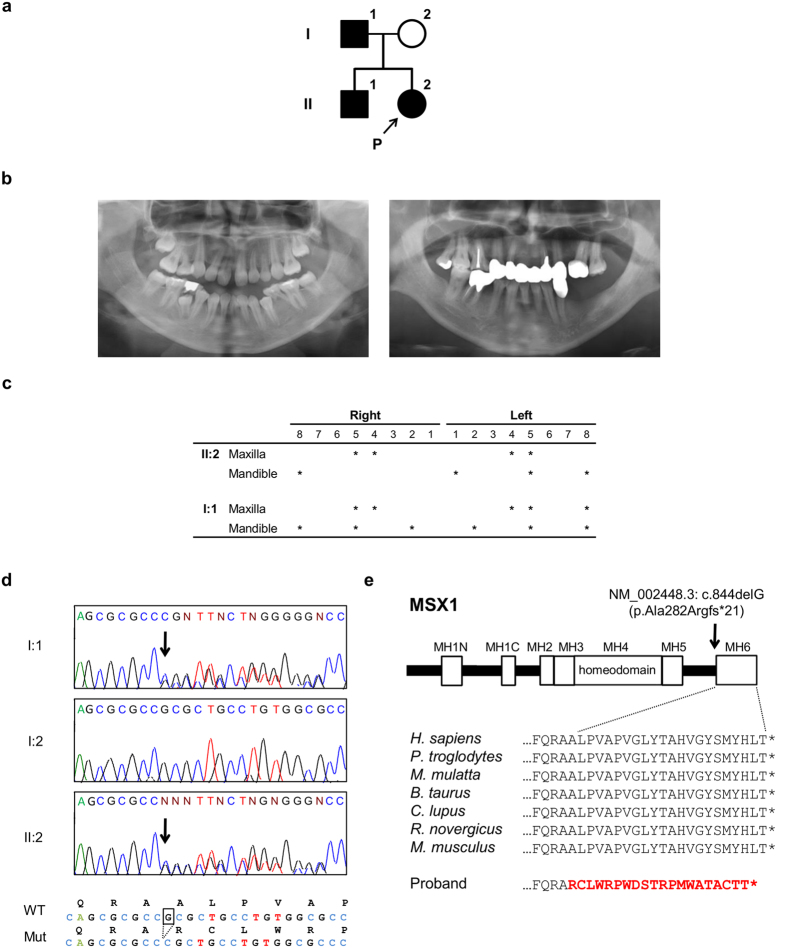
Identification of a heterozygous mutation in *MSX1* in a Japanese family with non-syndromic oligodontia. (**a**) Pedigree of affected (black filled symbols) father, son, and daughter (proband, P), and unaffected mother (open symbol). (**b**) Panoramic radiographs of the proband and her father. (**c**) Summary of permanent tooth agenesis in the proband and father (*affected tooth). (**d**) Chromatograms of *MSX1* segment in affected (I:1 and II:2) and unaffected (I:2) individuals. The arrow indicates frameshift mutation. DNA and corresponding amino-acid sequences of wild-type (WT) and mutant (Mut) alleles are shown below. (**e**) MSX1 domains (upper) and amino acid sequence conservation around MH6 in seven species (lower). Rectangles and the arrow represent MH domains and the location of the identified mutation (c.844delG, p.Ala282Argfs*21), respectively. Altered amino acid sequence in the proband is highlighted in red.

A family pedigree indicated autosomal-dominant inheritance of oligodontia (Fig. 1a). The proband (II:2), a 14-year-old girl, had agenesis of all maxillary first and second bicuspids, the mandibular left central incisor, and second bicuspid (Fig. 1b,c). Her father (II:1) had agenesis of all maxillary first and second bicuspids, the maxillary left third molar, the mandibular lateral incisors and second bicuspids, and both mandibular third molars (Fig. 1b,c); his mandibular left first and second molars had been extracted. Tooth agenesis of the proband’s brother was confirmed by interview only. No other physical anomalies, such as oral clefting or abnormalities of the nails, skin, hair, or sweat glands, were observed in I:1, II:1, or II:2.

### Mutation analyses

The proband was screened for mutations of genes previously related to tooth agenesis (i.e., *PAX9, AXIN2, EDA, WNT10A*, etc.) by targeted exome sequencing (TES), with next generation technology. A heterozygous nucleotide substitution in exon 4 of *WNT10A* (c.874 A>G, p.S292G) was detected, but not observed in family members by Sanger sequencing. This substitution had a 0.4% frequency in 1,208 sampled Japanese individuals [Human Genetic Variation Database (HGVD), http://www.genome.med.kyoto-u.ac.jp/SnpDB/], suggesting that it was an unrelated *de novo* mutation.

No other single nucleotide variations, small insertions and/or deletions (indels), or gross gains or losses were observed around other candidate genes. However, a segment of exon 2 in *MSX1* was covered by only a few TES reads. Sanger sequencing of this region revealed a heterozygous guanine deletion in exon 2 of *MSX1* (NM_002448.3: c.844delG) in the proband (II:2) and her affected father and brother (I:1, II:1), but not in her unaffected mother (Fig. 1d, data not shown). This deletion was predicted to cause a frameshift (p.A282Rfs*21) that alters 21 C-terminal amino acids starting from residue 282, creating a premature stop codon that results in a 301-, rather than 303-, residue truncated protein lacking MH6 domain (Fig. 1e). The sequence around MH6 is highly conserved in mammals (Fig. 1e).

MutationTaster (http://www.mutationtaster.org/) predicted that the detected deletion mutation would be disease-causing (probability = 0.999). It is not present in human genome variation databases [i.e., 1000 Genomes Project database (http://www.1000genomes.org/), NHLBI GO Exome Sequencing Project (ESP6500, http://evs.gs.washington.edu/EVS/), HGVD and integrative Japanese Genome Variation Database (iJGVD, https://ijgvd.megabank.tohoku.ac.jp/)] nor in disease-causing mutation databases [i.e., Human Gene Mutation Database Professional 2016.1 (HGMD, http://www.hgmd.org/) and ClinVar (http://www.ncbi.nlm.nih.gov/clinvar/)]. Hence, this *MSX1* mutation seems to be a novel oligodontia-causing genetic alteration inherited in an autosomal dominant manner.

### Generation of MH6-disrupted mice

**Figure 2:**
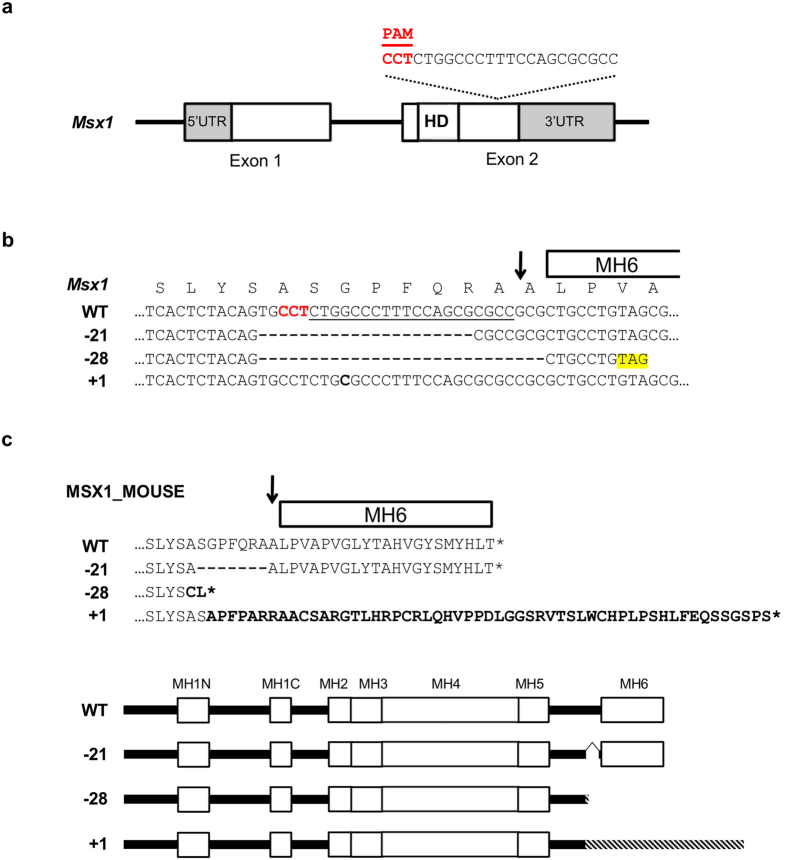
*Msx1* targeting using CRISPR/Cas system. (**a**) Targeted region in mouse *Msx1*. PAM sequence is highlighted in red. HD, homeodomain. (**b**) Sequences of the three mutant alleles observed in F_0_ animals and selected for further analyses. PAM sequence is highlighted in red in the wild-type (WT) sequence; the target sequence is underlined. The arrow indicates the guanine affected in the proband. Stop codon is highlighted in yellow (**c**) Amino-acid sequences and schematic representation of the wild-type (WT) and mutant MSX1 proteins. Striped areas indicate variant amino acids. Arrow indicates the conserved amino-acid affected in proband.

Most MSX1 variants isolated from patients with tooth agenesis to date involved single amino acid substitutions in the highly conserved homeodomain/MH4 sequence; few frameshift, nonsense, and splice-site mutations that lead to premature termination have been examined. To verify genotype-phenotype correlation *in vivo*, MH6 was targeted using CRISPR/Cas system in mice. A gRNA targeting upstream region of MH6 was designed and synthesized *in vitro* (Fig. 2a), and then co-injected with Cas9 mRNA into one-cell zygotes. Among the mosaic mice produced (17 of 20 F_0_), three mutant alleles were selected for phenotype analysis: in-frame 21-nucleotide deletion (*Msx1*^*−21*^) and frameshift 28-nucleotide deletion (*Msx1*^*−28*^) causing premature stop codon upstream of MH6; and single-nucleotide insertion (*Msx1*^*+1*^) causing a frameshift mutation affecting MH6 (Fig. 2b,c). The selected mice were mated to wild-type BDF1 mice.

### Secondary cleft palate and tooth agenesis in MH6 deficient mice

**Figure 3:**
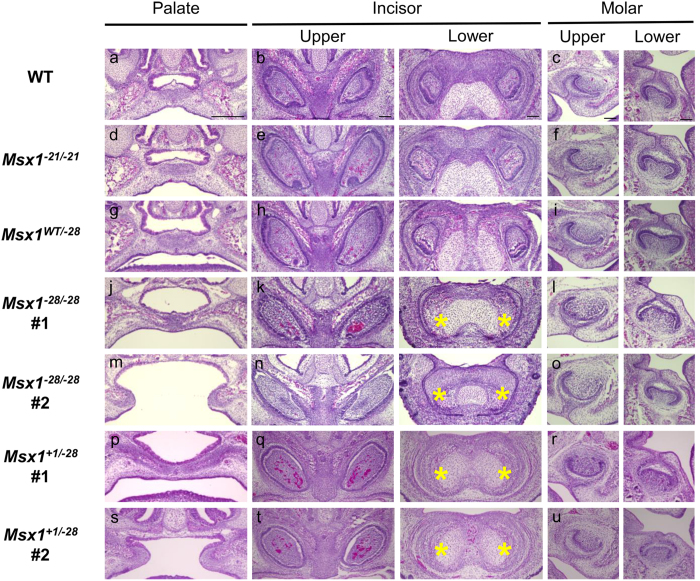
Coronal sections of *Msx1* mutant mice at E16.5. Samples are stained with hematoxylin and eosin. Asterisks indicate agenesis of lower incisors. Scale bars: 300 μm in the palate, 100 μm in the tooth bud.

**Figure 4:**
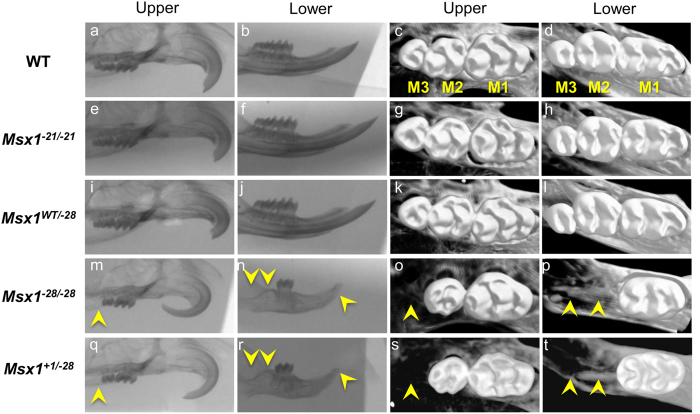
Micro-CT images of 4-week-old *Msx1* mutant mice. Arrowheads indicate tooth agenesis. M1, first molar; M2, second molar; and M3, third molar.

Because conventional *Msx1*-deficient mice is neonatally lethal^25^, phenotypes were first analyzed at E16.5. Coronal sections of *Msx*^*WT/−21*^and *Msx1*^*−21/−21*^ embryos showed normal tooth and palate development (Fig. 3a–f, data not shown). Although *Msx*^*WT/−28*^ mice showed normal palate and tooth development, two phenotypes were observed in *Msx1*^*−28/−28*^ mice: secondary cleft palate with agenesis of lower incisors; and agenesis of the lower incisors with a thin palate (Fig. 3g–o). Because one-third of the *Msx1*^*−28/−28*^ pups (5/15) exhibited palate development, phenotypes of 4-week-old mice were analyzed. All newborn homozygotes with cleft palates died neonatally, whereas those with a thin palate were viable. Micro-CT at 4 weeks old revealed no dentition anomalies in mice with in-frame homozygous mutations (Fig. 4e–h), but in homozygous *Msx1*^*−28/−28*^ mice, it revealed agenesis of the upper third molar and lower second and third molars as well as undersized upper second molars, in addition to the missing lower incisors observed at E16.5 (Fig. 4m–p). Similar phenotypes were observed in mice with compound heterozygous mutations affecting MH6 (Figs 3p–u and 4q–t).

## Discussion

In this study, we have reported a novel frameshift mutation in exon 2 of *MSX1* in a family with autosomal-dominant inheritance of non-syndromic oligodontia. The proband and her father had tooth agenesis phenotypes that were similar to those observed with previously identified *MSX1* mutations^26^,^27^. The guanine deletion identified (p.A282Rfs*21) is upstream of the MH6 domain and produced a frameshift that replaces the highly conserved MH6 sequence with an unrelated peptide. A heterozygous mutation disrupting the original stop codon and opening the reading frame was reported previously in a familial case of non-syndromic tooth agenesis^28^, but the presently identified mutation is the first to affect the entire MH6 domain.

Heterozygous MH6 disruption in model mice did not alter craniofacial development, whereas homozygous disruption resulted in agenesis of lower incisors with or without cleft palate at E16.5 as well as agenesis of the upper third molars and the lower second and third molars in 4-week-old mice. It is noteworthy that although the *MSX1* mutation in the proband family showed autosomal dominant inheritance, tooth agenesis and cleft palate were observed only in the bialellic disruption of MH6. Our findings are in line with previous studies reporting several nonsense oligodontia-causing mutations located in MH4 or its upstream regions with autosomal dominant trait^29^,^30^, although failure in tooth and palate development was inherited in a autosomal recessive manner in conventional *Msx1*-deficient mice with disrupted MH4 domain^25^.

The MH6 is the most C-terminal domain of MSX1, known as a PIAS binding domain^31^. It is necessary for localization of the MSX1 protein in the nuclear periphery, which enables repression of MyoD in myoblasts^31^. In addition, it has been reported that *Msx1* and *Msx2* act as potent transcriptional activators of the promoter of the Heat shock 70 kDa protein 1B gene *HSPA1B* through their C-terminal domains^32^. Although the MH6 domain has not been reported to interact with genes involved in craniofacial development, our results suggest that it is important in MSX1 function during tooth and palate development.

Most of the *MSX1* mutations that have been identified previously as causing non-syndromic tooth agenesis are located in MH4 domain, or in upstream regions that affect MH4^1^,^27^,^33^. The MH4 domain is involved in DNA binding and protein-protein interactions^34^,^35^. Functions of MH4-containing proteins are not mediated solely through protein-DNA interactions. It has been demonstrated that transcriptional repression by MSX1 occurs in the absence of DNA-binding sites; the repressor function is attributed to multiple domains in the N- and C-terminal regions of MSX1^36^. *In silico* analysis studies have indicated that missense mutations affecting MH4 in MSX1 alter the encoded 3D structures of the proteins, specifically by destabilizing the helix-turn-helix motif or altering its protein-DNA interactions^37^,^38^. Moreover *in vitro* studies have demonstrated that nuclear localization of MSX1 protein is mediated by the homeodomain^37^.

Finnerty *et al*. proposed that the non-random distribution of mutations in MH domains may be related to the yet unexplained genotype-phenotype correlation^12^. Specifically, they suggested that mutations that disrupt the MH1C or MH6 domain is associated with cleft disorders and may act through a dominant negative mechanism. On the other hand, tooth agenesis phenotypes attributed to MH1N and MH4 domain mutations have been explained by functional redundancy from the MSX2 domains, which are also highly conserved. Our findings contradict these observations, because the MH6 domain-affected members showed non-syndromic tooth agenesis phenotype.

Previously reported *Msx1-*deficient mice generated by MH4-disruptive insertion of the neomycin resistance gene exhibited cleft palate, alveolar bone deficiency in maxilla and mandible, bud stage arrest of tooth development, and other craniofacial abnormalities^25^. Unlike conventional *Msx1* deficient mice, our *Msx1*^*−28/−28*^ mice lacked only lower incisors, which were also absent in *Pax9;*^*+/-*^*Msx1*^*+/−*^ mice^39^. In addition, our observation of two different phenotypes in the palate of MH6-disrupted mice is consistent with previous findings. Jia *et al*. found that ~16% of *Bmp;*^*f/f*^*Wnt1Cre* mutant mice had cleft palate^40^ and Nakatomi *et al*. found that 39% of *Pax9;*^*−/−*^ *Msx1*^*−/−*^ mice were born with unilateral or bilateral cleft lip^39^.

This study has some limitations. First, we did not analyze possible off-target effects of the CRISPR/Cas9 modification. However, it is important to note that the genotype-phenotype correlations were maintained across mouse generations. Moreover, the phenotypes in MH6-disrupted mice were consistently observed in *Msx1*^*−28/−28*^ and *Msx*^*−1/+28*^. Second, although mouse models have been extensively used to understand tooth morphogenesis and to establish disease models, there are differences from humans in terms of number of the teeth. Mice have monophyodont dentition, presenting a single continuously growing incisor separated from three molars in each quadrant. In contrast, humans have dyphyodont dentitions with all four types of teeth. Therefore, further studies in non-human primates, such as marmosets in which premolars are observed, should be conducted.

In conclusion, we identified a novel mutation in a familial case of non-syndromic tooth agenesis affecting the MH6 domain of MSX1. Genotype-phenotype correlation was corroborated through CRISPR/Cas system-mediated gene targeting in mice. Thus, the present findings demonstrate that MH6 is functionally required in MSX1 for tooth and palate development.

## Methods

### Subjects

A Japanese family with tooth agenesis was enrolled. The pedigree was made based on clinical examination of the proband and interviews with all available family members. The diagnosis of oligodontia in the proband was verified by panoramic radiographs. Written informed consent was obtained from all participants. This study complied with the tenets of the Declaration of Helsinki and was approved by the ethics committee of Tokushima University Hospital (H24-8, H26-40).

### TES and Sanger sequencing

Genomic DNA was extracted from saliva samples with the Oragene^®^ DNA collection kit (OG-500, DNA Gentotec Inc., Ottawa, Canada), according to the manufacturer’s instructions. TES was performed with TruSight One sequencing panel (Illunima, San Diego, CA) and MiSeq benchtop sequencer (Illumina). Alignment of sequencing reads to a human reference genome (hg19), duplicate read removal, local realignment around indels, base quality score recalibration, variant calling, and detailed annotation were performed as described elsewhere^41^,^42^. Copy-number variations were detected relative to NGS data as described elsewhere^41^,^42^. Direct Sanger sequencing using polymerase-chain reaction products and BigDye Terminator v3.1 Cycle Sequencing Kit (Applied Biosystems, Foster City, CA, USA) were conducted with ABI 3500xL Genetic Analyzer (Applied Biosystems). Sequencing results were compared by BLAST (http://blast.ddbj.nig.ac.jp/top-j.html). Putative functional consequences of mutations were predicted *in silico* with MutationTaster.

### Animals

All animal experiments were approved by the Ethical Committee of Tokushima University for Animal Research (Approval number: 12064, T27–16). All procedures were conducted in accordance with the Guidelines for Animal Experiments of Tokushima University.

### Production of sgRNA and Cas9 mRNA

Two oligonucleotides (Forward, TAGGCGCGCTGGAAAGGGCCAG; Reverse, AAACCTGGCCCTTTCCAGCGCG) including the target sequence in *Msx1* were annealed and cloned into the BsaI site of the pDR274 plasmid (Addgene, Cambridge, MA). The Cas9 plasmid (pMLM3613, Addgene) and target sequence-inserted pDR274 were digested with PmeI and DraI, respectively. Linearized templates were transcribed with mMessage mMachine T7 ULTRA kits (Ambion, Austin, TX), and then treated with DNase I, according to the manufacturer’s instructions. Cas9-encoding mRNA and sgRNA were suspended in appropriate volumes of RNase-free water, after purification by phenol-chloroform-isoamylalcohol extraction and isopropanol precipitation.

### RNA microinjection into embryos

Cas9-encoding mRNA (10 ng/μl) and sgRNA (1 ng/μl) quantified by a NanoDrop 2000 UV-Vis spectrophotometer (Thermo Fisher Scientific Inc., Wilmington, DE) were co-injected into the cytoplasm of fertilized eggs, obtained after mating BDF1 (C57BL/6 × DBA2 F_1_) male with superovulated female mice. Injected eggs were cultured overnight in M16 medium (Sigma, St. Louis, MO) at 37 °C in 5% CO_2_. Two-cell embryos were then transferred into the oviducts of pseudopregnant MCH(ICR) mice.

### Mouse genotyping

To verify CRISPR/Cas9-mediated mutations, genomic DNA was extracted from tail biopsies. The genomic regions flanking the gRNA target were PCR amplified with KOD-Plus-Neo (Toyobo, Osaka, Japan) and an *Msx1*-specific primer pair (Forward, 5′-CGCAAGCACAAGACTCTCTTT-3′; and Reverse 5′-AGGGGTCAGATGAGGAAGGT-3′), according to the manufacturer′s instructions. PCR products were purified for direct or cloned sequencing using Wizard SV Gel and PCR Clean-up System (Promega). For mosaic F_0_ genotyping, purified PCR amplicons were cloned into plasmids using DynaExpress TA PCR Cloning Kits (BioDynamics Laboratory, Tokyo, Japan). Isolated plasmids from each sample were sequenced with a BigDye Terminator Sequence Kit ver. 3.1 and an ABI 3500xL Genetic Analyzer (Applied Bioystems). After targeting verification, F_0_ mice were mated with wild-type BDF1 mice to propagate alleles of interest. F_2_ to F_4_ generations were analyzed.

### Histology and Micro-computed tomopraphy (Micro-CT)

Dissected samples at E16.5 were immerse-fixed immediately in 4% paraformaldehyde, dehydrated in ethanol, and paraffin-processed for sectioning. Serial coronal sections (7 μm) were stained with hematoxylin and eosin.

Maxillae and mandibles of 4-week-old mice were resected and fixed overnight in 70% ethanol after removal of soft tissues. Molars and incisors were analyzed by high-resolution micro-CT (Skyscan 1176, operated at 50 kV and 200 μA; Bruker-microCT, Kontich, Belgium). Two-dimensional images were used to generate three-dimensional (3D) renderings with CTVox 3D Creator software (version 3.0, Bruker). The resolution of the micro-CT images was 9 μm per pixel.

## Additional Information

**How to cite this article**: Mitsui, S. N. *et al*. Novel human mutation and CRISPR/Cas genome-edited mice reveal the importance of C-terminal domain of MSX1 in tooth and palate development. *Sci. Rep.* **6**, 38398; doi: 10.1038/srep38398 (2016).

**Publisher's note:** Springer Nature remains neutral with regard to jurisdictional claims in published maps and institutional affiliations.
